# Hashimoto Thyroiditis, Anti-Parietal Cell Antibodies: Associations With Autoimmune Diseases and Malignancies

**DOI:** 10.3389/fendo.2022.860880

**Published:** 2022-04-22

**Authors:** Georgios Boutzios, Eleni Koukoulioti, Andreas V. Goules, Ioannis Kalliakmanis, Ilias Giovannopoulos, Panayiotis Vlachoyiannopoulos, Haralampos M. Moutsopoulos, Athanasios G. Tzioufas

**Affiliations:** ^1^ Department of Pathophysiology, School of Medicine, National and Kapodistrian University of Athens, Athens, Greece; ^2^ Endoscopy Unit, Ygeia Hospital AE, Athens, Greece; ^3^ Academy of Athens, Athens, Greece

**Keywords:** Hashimoto thyroiditis, anti-parietal cell antibodies, organ-specific autoimmunity, systemic autoimmunity, gastric malignancies

## Abstract

**Background:**

Hashimoto thyroiditis (HT) is an autoimmune disease which may result in extensive damage of the thyroid gland. Chronic atrophic gastritis (CAG), is the most frequent HT-associated disorder, with anti-parietal cell autoantibodies (APCA) being a screening test for autoimmune CAG. The aim of this study was to investigate, in a cohort of HT patients: a) the prevalence of APCA in an attempt to define their clinical phenotype and b) any possible associations of APCA with other autoimmune diseases and malignancies.

**Methods:**

This is a single-center, case-control study, conducted at a University Hospital. The study included patients with HT diagnosed between November 2017 and November 2020. Excluded were patients <18 years old, with sonographic features of HT but negative thyroid peroxidase (TPOAbs) or thyroglobulin autoantibodies (TgAbs), Graves’ disease, Down or Turner’s syndrome.

**Results:**

A total of 840 patients with HT were included in the study, from whom 180 (21.4%) had positive APCA. A total of 79 patients (9.4%) had one or more organ-specific autoimmune diseases and 61 (7.3%) had a systemic autoimmune disease. Autoimmune diseases were more frequent in female than in male patients (17.9% versus 10.9%, p = 0.05). APCA-positive patients were older than APCA-negative (54.1 ± 13.5 versus 49.0 ± 14.6, p <0.001) and had more often positive TPOAbs (93.3% versus 83.9%, p=0.001). Gastric neoplasms were documented only in APCA-positive patients (p <0.001). A higher frequency of organ-specific autoimmune diseases was observed in the APCA-positive group (14.4% versus 8%, p = 0.024). In the subgroup of patients with additional autoimmune diseases (n = 140), younger age and positive APCA were independently associated with the presence of organ-specific autoimmunity (OR 0.954, 95% CI 0.927–0.982 and OR 3.100, 95% CI 1.256–7.652, respectively). Papillary thyroid cancer (PTC) occurred in 3.5% of patients (26/29 women). Positive family history for thyroid autoimmunity and negative TPOAbs were the only independent risk factors for PTC among women (OR 3.228, 95% CI 1.173–8.887 and 0.315, 95% 0.113–0.881, respectively).

**Conclusion:**

This study reveals for the first time an association of APCA with organ-specific autoimmunity in HT patients. APCA together with patient age were independently associated with the presence of organ-specific autoimmunity. Finally, this study showed an association between APCA and gastric neoplasms in these patients.

## Introduction

Hashimoto thyroiditis (HT) is an organ-specific autoimmune disease, characterized by the immune-mediated destruction of the thyroid gland and together with Graves’ disease, constitutes the functional ends of autoimmune thyroid disease (AITD) spectrum. HT is the most common autoimmune disease with a prevalence in the general population ranging between 10 and 12% (1). The prevalence is higher in women than in men and increases with age (1). The presence of autoantibodies against thyroid peroxidase (TPOAbs) and thyroglobulin (TgAbs), in addition to hypo-echogenicity on thyroid ultrasound are considered the hallmarks for HT diagnosis (1). Coexistence with other organ-specific or systemic autoimmune disorders can be found in 14.3% of HT patients (2). The most prevalent autoimmune disorders in patients with HT are rheumatoid arthritis, chronic atrophic gastritis (CAG) of autoimmune origin, systemic lupus erythematosus, Sjögren’s syndrome, Addison’s disease, celiac disease, and non-segmental vitiligo (2–4).

Interestingly, the thyroid gland and the stomach, share morphologic, biochemical, functional, and immunologic properties, most likely due to their common embryologic origin (5). Anti-parietal cell antibodies (APCA) directed against the proton pump H^+^/K^+^ ATP-ase, is a screening test for autoimmune chronic atrophic gastritis and gastric enterochromaffin-like (ECL) cell hyperplasia, and can be detected in 85–90% of patients with pernicious anemia (6). APCA-positive patients gradually develop asymptomatic atrophy of gastric mucosa and parietal cells, and those with pernicious anemia display a 6.8% risk of gastric carcinoma and carcinoid tumors (7, 8). Hughes et al. reviewed data from a small number of patients with pernicious anemia who underwent esophagogastroduodenoscopy or gastrectomy and found premalignant or malignant lesions in 76.5% of these patients, and an increased incidence of gastric neuroendocrine neoplasms (GNENs) (17.7%) and infrequently adenocarcinoma (2.9%) (9). Furthermore, previous studies have shown a possible association of AITD with thyroid carcinoma (10). In the light of previous knowledge, we investigated in a cohort of HT patients: a) the prevalence of APCA positivity in an attempt to define their clinical phenotype and b) any potent association of APCA with other autoimmune diseases and malignancies.

## Material and Methods

This is a single-center cross-sectional case-control study, conducted in the autoimmune endocrinopathies outpatient clinic of the Department of Pathophysiology, at the Laikon General Hospital, Medical School, National and Kapodistrian University of Athens. All patients diagnosed with HT between November 2017 and November 2020 were initially evaluated for the study. The HT diagnostic criteria included positive TPOAbs and/or TgAbs (1). All patients were systematically screened for the presence of APCA and for laboratory findings of anemia.

Patients <18 years old, with sonographic features of HT but negative TPOAbs or TgAbs, Graves’ disease positive for TPOAbs and/or TgAbs, Down syndrome or Turner’s syndrome, and also those who had no assessment of APCA were excluded from the current study. Clinical data, namely, family history and medical history for thyroid disease, GNENs, gastric and other malignancies and organ-specific or systemic autoimmune diseases were recorded in all included patients. Evaluation of systemic autoimmune rheumatic diseases included physical examination, laboratory and immunologic profile and review of medical charts by a single rheumatologist according to the ACR/EULAR classification criteria (https://www.eular.org/recommendations_eular_acr.cfm, assessed on April 8, 2021). All APCA-positive patients underwent gastroscopy and gastric biopsy to confirm histologically atrophy of parietal cells and to document the presence of pre-malignant or malignant lesions (11). In the HT patients the clinical, laboratory and immunologic data and potential associations with systemic or organ-specific autoimmunity and malignancies of APCA positive vs APCA-negative patients were evaluated. The study was approved by the local ethical committee after obtaining informed consent from the patients and in compliance with general data protection regulations (GDPR) of the European Union. All procedures involving human participants were performed in the context of standard of care according to the judgment of the physicians and in accordance with the ethical standards of the institutional and/or national research committee and with the 1964 Helsinki declaration and its later amendments or comparable ethical standards.

### Assays

TPOAbs were assessed with a chemiluminometric Immunoassay test kit (normal values: <34 iu/ml). TgAbs were assessed with an electrochemiluminescence immunoassay. The Roche E170 TgAbs is a competitive assay in which patient TgAbs competes for binding to solid-phase Tg with ruthenylated human monoclonal TgAbs. Interassay precision was 5.4% at 80.8 kU/L and 5.9% at 238.6 kU/L (normal values.: <115 iu/ml). APCA were assessed with an immunofluorescence assay (IFA) using mouse stomach substrate. Specific immunofluorescence pattern at serum dilutions >1/80 defined the positive values.

### Statistical Analysis

A comparison between the APCA-positive vs APCA-negative subgroups was carried out using 2-sided Chi-squared test corrected by Fisher’s exact test when appropriate for categorical variables, while the continuous variables were analyzed by Student’s t-test for parametric and Mann–Whitney U test for non-parametric continuous variables. Among patients with additional autoimmune diseases, a bivariate logistic regression analysis was performed to identify factors associated with organ-specific autoimmunity and the unadjusted ORs and their 95% Confidence Intervals are presented. The independent variables of the model comprise of age, gender, presence of APCA, TPOAbs and TgAbs, family history for thyroid autoimmunity, and presence of malignancy. Moreover, a bivariate logistic analysis was performed to identify factors associated with PTC and overall malignancies (dependent variables). In these models, independent variables were age, gender, presence of APCA, TPOAbs and TgAbs, family history for thyroid autoimmunity and coexistence of other autoimmune diseases. A p-value <0.05 was considered statistically significant. SPSS software (SPSS 25. Inc. Chicago, IL) was used for the statistical analysis.

## Results

### Cohort Characteristics

Overall, 840 of the total 1,126 patients with HT, agreed and were included in the study, from whom 180 (21.4%) had positive APCA. No HT included patient had laboratory findings of anemia. **Table 1** shows the clinical characteristics of the patients included in the study. Men did not differ from women in terms of age, presence of TPOAbs, TgAbs, and APCA, family history for thyroid autoimmunity and malignancies (data not shown). However, underlying autoimmune diseases were more frequent in female than in male patients (17.9% versus 10.9%, p = 0.05) independently of other co-variants (OR 1.840, 95% CI 1.037–3.264). After classifying autoimmune diseases as either systemic (e.g., systemic lupus erythematosus, rheumatoid arthritis, and Sjögren’s syndrome) or organ-specific, if a particular organ or tissue was preferentially targeted by the immune system, (e.g., type 1 diabetes mellitus and vitiligo), it was found that 79 patients (9.4%) had one or more organ-specific autoimmune diseases and 61 (7.3%) fulfilled criteria for a systemic autoimmune disease. Among 79 patients with organ-specific autoimmunity, 73 (8.7%) had one additional affected organ besides HT, 5 (0.6%) two organs and 1 (0.01%) three organs. A list of the observed autoimmune diseases is provided in the supplementary material (**Supplementary Table 1**). The most common systemic autoimmune disease was rheumatoid arthritis (2.1%) followed by systemic lupus erythematosus (1.4%), while the most common organ-specific autoimmunity was non-segmental vitiligo (3.0%) followed by diabetes mellitus type 1 (1.2%).

**Table 1:** Clinical characteristics of APCA-negative and APCA-positive patients with HT (n=840).

Characteristics	All patients (n = 840)	APCA negative (n = 660)	APCA positive (n = 180)	p value*
**Mean age (years)**	50.1±14.5	49.0±14.6	54.1±13.5	**<0.001**
**Gender (female)**	693 (82.5%)	550 (83.3%)	143 (79.4%)	0.268
**TPO antibodies**	717/835 (85.9%)	503 (83.9%)	156 (93.3%)	**0.001**
**Tg antibodies**	578/824 (70.1%)	454 (70.2%)	124 (70.1%)	1.000
**Coexistence of other autoimmune diseases**	140 (16.7%)	104 (15.8%)	36 (20.0%)	0.214
** no (thyroid only)**	700 (83.3%)	556 (84.2%)	144 (80%)	**0.024**
** Organ-specific autoimmunity**	79 (9.4%)	53 (8%)	26 (14.4%)	
** Systemic autoimmunity**	61 (7.3%)	51 (7.7%)	10 (5.6%)	
**Family history for thyroid autoimmunity**	461/835 (55.2%)	361 (54.9%)	100 (56.2%)	0.799
**Malignancy**	85 (10.1%)	63 (9.5%)	22 (12.2%)	0.328
** PTC**	29 (3.5%)	20 (3.0%)	9 (5.0%)	0.246
** GNEN**	4 (0.5%)	0	4 (2.2%)	**<0.001**
** Gastric Ca**	1 (0.1%)	0	1 (0.6%)	
** Breast Ca**	21 (3%^#^)	19 (3.5%^#^)	2 (1.4%^#^)	0.196
** Other**	30 (3.6%)	24 (3.6%)	6 (3.3%)	

*P value refers to comparisons between APCA-negative and APCA-positive patients; ^#^among women computed; APCA, anti-parietal cell antibodies; TPO, thyroid peroxidase; Tg, thyroglobulin, PTC, papillary thyroid carcinoma; GNEN, gastric neuroendocrine neoplasm; Ca, carcinoma. Bold values denote statistical significance at the p<0.05 level.

### Comparisons Between APCA-Positive and APCA-Negative Patients

The APCA-positive patients were older than the APCA-negative patients (54.1 ± 13.5 versus 49.0 ± 14.6, p <0.001) and had more often positive TPOAbs (93.3% versus 83.9%, p = 0.001). No differences were observed regarding gender, presence of TgAbs, family history for thyroid autoimmunity or presence of other autoimmune diseases or malignancies between the two groups (**Table 1**). No gastric neoplasms were observed among the APCA-negative patients, whereas four GNENs and one gastric adenocarcinoma were diagnosed in the group of APCA-positive patients (p <0.001). Among the APCA-negative patients only 8% had an additional organ-specific autoimmune disease compared to 14.4% in the APCA-positive group. Conversely, 7.7% in the APCA-negative group had a systemic autoimmune disease compared to 5.6% in the APCA-positive group (p = 0.024).

### Comparisons in the Subgroup of Patients With Additional Autoimmune Disease

We subsequently analyzed patients with coexistence of other autoimmune diseases (n = 140). Distribution of gender, TPOAbs, TgAbs, presence of family history for thyroid autoimmunity and malignancies did not differ significantly between patients with organ-specific and those with systemic autoimmunity (**Table 2**). Patients with organ-specific autoimmune diseases were younger (48.7 ± 12.5 years versus 55.2 ± 13.9, p = 0.005). Moreover, APCA were more often positive among patients with organ-specific autoimmune diseases than those with systemic diseases (32.9% versus 16.4%, p = 0.034). In the multivariate analysis, younger age and positive APCA were the only independent risk factors for the presence of organ-specific autoimmunity (OR 0.954, 95% CI 0.927–0.982, and OR 3.100, 95% CI 1.256–7.652, respectively).

**Table 2:** Univariate and multivariate analysis of factors associated with organ-specific and systemic autoimmunity in HT patients (n = 140).

Characteristics	Systemic autoimmunity (n = 61)	Organ-specific autoimmunity (n = 79)	Univariate analysis (p value)	Multivariate analysis^#^ (OR, 95% CI)
**Mean age (years)**	55.20±13.9	48.72±12.5	**0.005**	**0.954 (0.927-0.982), p = 0.002**
**Gender (females)**	55 (90.2%)	69 (87.3%)	0.790	0.737 (0.229-2.374), p = 0.610
**APCA**	10 (16.4%)	26 (32.9%)	**0.032**	**3.100 (1.256-7.652), p = 0.014**
**TPO antibodies**	51 (83.6%)	67 (84.8%)	1.000	1.214 (0.422-3.490), p = 0.719
**Tg antibodies**	42/59 (71.2%)	59/78 (75.6%)	0.695	1.879 (0.776-4.552), p = 0.162
**Family history for thyroid autoimmunity**	32 (52.5%)	50/77 (64.9%)	0.164	1.723 (0.807-3.680), p = 0.160
**Malignancies**	5 (8.2%)	5 (6.3%)	0.747	1.110 (0.265-4.651), p = 0.886

^#^135 patients included due to missing values.

Nagelkerke R Square 0.184, Cox & Schnell R Square 0.137 for logistic regression analysis.

APCA, anti-parietal cell antibodies; TPO, thyroid peroxidase; Tg, thyroglobulin, OR, odds ratio; 95% CI, 95% confidence intervals. Bold values denote statistical significance at the p<0.05 level.

### Papillary Thyroid Carcinoma (PTC) and Other Malignancies in HT Patients

In our cohort, 29 (3.5%) patients of whom 26 (89.7%) were women, were diagnosed with PTC at a mean age of 47.9 ± 17.0 years old. Nine of them had positive APCA (31%). There was no statistically significant superiority of APCA in patients with PTC compared to those without (31% versus 21.1%, p = 0.246, **Table 3**). Furthermore, in the univariate analysis PTC was not associated with age, gender, TPOAbs, TgAbs, family history for thyroid autoimmunity or coexistence of other autoimmune diseases. Patients with PTC tend to be younger than 40 years (41.4% versus 24.5%, p = 0.049, **Table 3**). The multivariate analysis showed that the presence of positive family history for thyroid autoimmunity was independently associated with a 2.8-fold increased risk of PTC (**Table 3**). Because the majority of PTC patients were women, we conducted a separate analysis excluding the three male patients. In the univariate analysis, women with PTC had less frequently positive TPOAbs than those without PTC (69.6% versus 85.6%, p = 0.043). In the multivariate analysis, a positive family history for thyroid autoimmunity was associated with a 3.2-fold increased risk of PTC (OR 3.228, 95% CI 1.173–8.887), whereas patients with positive TPOAbs had a 69% decreased risk of PTC (OR 0.315, 95% 0.113–0.881, see **Supplementary Table 2**).

**Table 3:** Univariate and multivariate analysis of factors associated with papillary thyroid carcinoma in HT patients (n = 840).

Characteristics	With PTC (n = 29)	Without PTC (n = 811)	Univariate analysis (p value)	Multivariate analysis^#^ (OR, 95% CI)
**Age (years)**	47.9±17.0	50.2±14.5	0.414	–
**Age (≥40 vs <40 years)**	17 (58.6%)	612 (75.5%)	**0.049**	0.463 (0.202-1.062)
**Gender (females)**	26 (89.7%)	667 (82.2%)	0.340	1.823 (0.530-6.276)
**APCA**	9 (31.0%)	171 (21.1%)	0.246	1.906 (0.747-4.864)
**TPO antibodies**	19/26 (73.1%)	698/809 (86.3%)	0.079	0.440 (0.164-1.178)
**Tg antibodies**	23 (79.3%)	555/795 (69.8%)	0.309	1.214 (0.451-3.267)
**Family history for thyroid autoimmunity**	21 (72.4%)	440/806 (54.6%)	0.086	**2.808 (1.105-7.134)**
**Coexistence of other autoimmune diseases**	2 (6.9%)	138 (17.0%)	0.205	0.368 (0.085-1.592)

^#^814 patients included due to missing values.

Nagelkerke R Square 0.079, Cox & Schnell R Square 0.019 for logistic regression analysis.

APCA, anti-parietal cell antibodies; TPO, thyroid peroxidase; Tg, thyroglobulin, PTC, papillary thyroid carcinoma; OR, odds ratio; 95% CI, 95% confidence intervals. Bold values denote statistical significance at the p<0.05 level.

Overall, 85 patients had been diagnosed with malignant disease in our cohort (87.1% women, mean age 54.2 ± 15.4 years). The presence of malignancy was not associated with gender, TPOAbs, TgAbs, APCA, family history for thyroid autoimmunity or coexistence of other autoimmune diseases, but individuals with a malignant disease were older than those without (54.2 ± 15.4 versus 49.6 ± 14.4 years, p = 0.006). In multivariate analysis, only age was significantly associated with the presence of malignant disease (OR 1.024, 95% CI 1.007–1.042, see **Supplementary Table 3**).

## Discussion

APCA antibodies are detected frequently among HT patients and APCA-positive patients had more often positive TPOAbs, but no differences were observed regarding TgAbs, implying potentially shared epitopes between APCA and TPO, as suggested by previous studies (12), although no specific role of thyroid autoantibodies in the pathogenesis of CAG has been so far documented (13). APCA and patient age were independently associated with the presence of organ-specific autoimmunity. In particular, patients with positive APCA had a 3.1-fold higher probability of having an additional organ-specific autoimmune disease.

The prevalence of APCA in our cohort is lower compared to that reported by Checchi et al. in a large Italian cohort, namely, 2,275 patients with HT. This difference can probably be attributed to the younger age of patients included in our study (14). Other studies conducted in the last two decades in adults have reported a prevalence of APCA ranging from 16.3 to 40%, depending on the number, age and ethnicity of included patients (13, 15–19).

Notably, no patient had laboratory findings of anemia in our cohort, although anemia is reported to some extent in patients with chronic atrophic gastritis either due to *Helicobacter pylori* infection or of autoimmune origin (20, 21). This can be attributed to the relatively young age of patients included in our study, since the prevalence of CAG increases with age (22). In addition, patients in our cohort were screened for APCA because the diagnosed HT and endoscopy was performed only if APCA were found positive and not because of clinical manifestations of anemia. This is a crucial difference in comparison with previous studies reporting hematological complications of chronic atrophic gastritis, where gastroscopies were performed due to gastrointestinal complaints and/or findings of anemia.

Gastric neoplasms were reported only in APCA-positive patients (p <0.001). Because of the small number of gastric malignancies in our cohort, we could not perform a multivariate analysis, and we were not able to assess the severity of gastric atrophy by histology. Studies regarding the risk of gastric neoplasms in patients with CAG do not discriminate between CAG due to chronic *H. pylori* infection and CAG of autoimmune origin, and large prospective studies assessing the overall risk of gastric cancer and neuroendocrine neoplasms in APCA-positive CAG are lacking (7, 23).

PTC occurring in 3.5% of the patients with negative TPOAbs was the only independent risk factor for PTC among women (Odds Ratios 3.228, 95% CI 1.173–8.887 and 0.315, 95% 0.113–0.881, respectively) (**Supplementary Table 2**) (24, 25). The correlation of PTCs with the absence of TPOAbs connotes most likely an epidemiologic rather than a pathogenetic association, since the evaluation of HT patients was performed at the time of HT diagnosis when it is more likely to detect TgAbs than TPOAbs which occur later during the HT course. Thus, the association of PTC with the absence of TPOabs implies that PTC diagnosis was a late sequel in the course of HT.

In our cohort, thirty-one (3.7%) patients had overall 3 or more organs affected. The association of autoimmune thyroiditis with other autoimmune disorders is well established, and the most frequent associations are autoimmune thyroiditis with CAG and non-segmental vitiligo, and autoimmune thyroiditis with CAG and polymyalgia rheumatica, respectively (26). Autoimmune thyroid diseases also tend to cluster within families, with 40 to 50% of patients reporting another family member with a thyroid disorder (2, 27). Similarly, 55.2% of patients in our cohort had a positive family history for thyroid autoimmunity. Possible associations of APCA with the presence of other autoimmune diseases in patients with autoimmune thyroid diseases have been investigated in previous publications. In the study by Utiyama et al., the frequency of coexisting autoimmune diseases in 107 patients with HT did not differ between APCA-positive and APCA-negative patients (15). Similarly, we did not find a different prevalence of coexisting autoimmune diseases between APCA-positive and APCA-negative patients in our cohort. However, we found a difference concerning the type of autoimmune diseases, i.e., organ-specific or systemic. In fact, we observed a higher frequency of organ-specific autoimmune diseases in the APCA-positive group (14.4% versus 8%, p = 0.024), which was a very interesting finding, that has not been reported so far. Therefore, we analyzed separately the group of patients with coexistence of other autoimmune diseases (n = 140). In the multivariate analysis, only APCA together with patient age were independently associated with the presence of organ-specific autoimmunity. In particular, patients with positive APCA had a 3.1-fold higher probability of suffering from an additional organ-specific autoimmune disease, most commonly CAG of autoimmune origin. In this case, genetic susceptibility, shared epitopes or epitope spreading and loss of immunoregulatory mechanisms in various tissues, may contribute in clustering of organ-specific autoimmune responses (5, 12).

A limitation of our study is that APCA were measured by IFA, which is a semi-quantitative and qualitative method and not by ELISA, which would have eventually allowed quantitative assessments and correlations. Nevertheless, the two methods are comparable and monitoring of APCA levels was not a purpose of our study (28, 29).

A strength of our study is the large number of HT patients included and the exclusion of patients with Grave’s disease, which may have a different pathogenetic basis.

In conclusion, our study reveals for the first time an association of APCA with organ-specific autoimmunity in HT patients. APCA together with patient age were independently associated with the presence of organ-specific autoimmunity. Moreover, our study showed an association between APCA and gastric neoplasms in these patients.

## Data Availability Statement

The original contributions presented in the study are included in the article/**Supplementary Material**. Further inquiries can be directed to the corresponding author.

## Ethics Statement

The studies involving human participants were reviewed and approved by the Laiko General Hospital and School of Medicine National and Kapodistrian University of Athens. The patients/participants provided their written informed consent to participate in this study.

## Author Contributions

GB conceived the idea of the study, designed the study and wrote the manuscript. EK participated in the design of the study, wrote the manuscript and performed the statistical analysis. AG reviewed and edited of the manuscript. IK and IG were responsible for data collection. PV reviewed and edited the manuscript. HM provided revisions to the scientific content of the manuscript. AT reviewed, edited and provided revisions to scientific content of the manuscript. All authors listed have made a substantial, direct, and intellectual contribution to the work and approved it for publication.

## Author Disclaimer

All claims expressed in this article are solely those of the authors and do not necessarily represent those of their affiliated organizations, or those of the publisher, the editors and the reviewers. Any product that may be evaluated in this article or claim that may be made by its manufacturer is not guaranteed or endorsed by the publisher.

## Conflict of Interest

The authors declare that the research was conducted in the absence of any commercial or financial relationships that could be construed as a potential conflict of interest.

## Publisher’s Note

All claims expressed in this article are solely those of the authors and do not necessarily represent those of their affiliated organizations, or those of the publisher, the editors and the reviewers. Any product that may be evaluated in this article, or claim that may be made by its manufacturer, is not guaranteed or endorsed by the publisher.
