# Supplementary material for: Hashimoto Thyroiditis, Anti-Parietal Cell Antibodies: Associations With Autoimmune Diseases and Malignancies
Source: Front Endocrinol (Lausanne). 2022 Apr 22;13:860880. doi: 10.3389/fendo.2022.860880 (PMC9072778; doi:10.3389/fendo.2022.860880)
Supplement: Supplementary file 1 [file Presentation_1.pdf]

Supplementary Table 1 Type and frequency of the autoimmune diseases observed among patients with Hashimoto's thyroiditis

Autoimmune disease	Number of patients (%)
**Vitiligo**	25 (3.0)
**Rheumatoid arthritis**	18 (2.1)
**Systemic lupus erythematosus**	12 (1.4)
**Multiple sclerosis**	12 (1.4)
**Diabetes mellitus type 1**	10 (1.2)
**Sjögren's disease**	9 (1.1)
**Psoriatic arthritis**	9 (1.1)
**Premature ovarian failure**	9 (1.1)
**Systemic scleroderma**	6 (0.7)
**Inflammatory bowel diseases (Colitis ulcerosa/Crohn's disease)**	6 (0.7)
**Psoriasis**	6 (0.7)
**Addison's disease**	6 (0.7)
**Celiac disease**	5 (0.6)
**Idiopathic thrombocytopenic purpura**	4 (0.5)
**Autoimmune liver diseases (primary biliary cholangitis, autoimmune hepatitis)**	3 (0.4)
**Alopecia areata**	3 (0.4)
**Glomerulonephritis**	2 (0.3)
**Polymyalgia rheumatica**	2 (0.3)
**Mixed connective tissue disease**	1 (0.1)
**Lichen planus**	1 (0.1)
**Dermatomyositis**	1 (0.1)
**Myasthenia gravis**	1 (0.1)
**Vasculitis**	1 (0.1)
**Ankylosing spondylitis**	1 (0.1)
**Seronegative arthritis**	1 (0.1)
**Autoimmune hemolytic anemia**	1 (0.1)
**Pemphigus vulgaris**	1 (0.1)
**Schoenlein Henoch purpura**	1 (0.1)
**Myelitis transversa**	1 (0.1)
**Latent autoimmune diabetes in adults**	1 (0.1)
**Autoimmune polychondritis**	1 (0.1)
**CREST syndrome**	1 (0.1)

Supplementary Table 2 Univariate and multivariate analysis of factors associated with papillary thyroid carcinoma among women

Characteristics	With PTC	Without PTC	Univariate analysis (p value)	Multivariate analysis (OR, 95% CI)
**Age (years)**	48.9±17.4	50.2±14.0	0.624	1.001 (0.971-1.032)
**APCA**	8 (30.8%)	135 (20.2%)	0.215	1.680 (0.616-4.586)
**TPO antibodies**	16 (69.6%)	569 (85.6%)	**0.043**	**0.315 (0.113-0.881)**
**Tg antibodies**	20 (76.9%)	462 (70.6%)	0.523	0.867 (0.308-2.439)
**Family history for thyroid autoimmunity**	19 (73.1%)	365 (55.0%)	0.073	**3.228 (1.173-8.887)**
**Coexistence of other autoimmune diseases**	2 (7.7%)	122 (18.3%)	0.201	0.404 (0.093-1.761)
**Nagelkerke R Square 0.071, Cox & Schnell R Square 0.018 for logistic regression analysis<br>APCA, anti-parietal cell antibodies; TPO, thyroid peroxidase; Tg, thyroglobulin, PTC, papillary thyroid carcinoma; OR, odds ratio; 95% CI, 95% confidence intervals**				

Supplementary Table 3 Univariate and multivariate analysis of factors associated with malignancies

Characteristics	With malignancy	Without malignancy	Univariate analysis (p value)	Multivariate analysis (OR, 95% CI)
**Age (years)**	54.2±15.4	49.6±14.4	**0.006**	**1.024 (1.007-1.042)**
**Gender (females)**	74 (87.1%)	619 (82.0%)	0.293	1.363 (0.696-2.669)
**APCA**	22 (25.9%)	158 (20.9%)	0.328	1.124 (0.641-1.973)
**TPO antibodies**	67 (82.7%)	650 (86.2%)	0.401	0.682 (0.354-1.316)
**Tg antibodies**	59 (71.1%)	519 (70.0%)	0.900	0.934 (0.547-1.595)
**Family history for thyroid autoimmunity**	48 (56.5%)	413 (55.1%)	0.819	1.212 (0.752-1.954)
**Coexistence of other autoimmune diseases**	10 (11.8%)	130 (17.2%)	0.223	0.654 (0.326-1.313)
**Nagelkerke R Square 0.030, Cox & Schnell R Square 0.014 for logistic regression analysis<br>APCA, anti-parietal cell antibodies; TPO, thyroid peroxidase; Tg, thyroglobulin; OR, odds ratio; 95% CI, 95% confidence intervals**				
